# Efficacy of Tumor-Infiltrating Lymphocytes Combined with IFN-*α* in Chinese Resected Stage III Malignant Melanoma

**DOI:** 10.1155/2017/1092507

**Published:** 2017-08-20

**Authors:** Wei Li, Linping Xu, Yaomei Wang, Lingdi Zhao, Daniel B. Kellner, Quanli Gao

**Affiliations:** ^1^Department of Immunotherapy, The Affiliated Cancer Hospital of Zhengzhou University, Henan Cancer Hospital, Zhengzhou, Henan 450008, China; ^2^School of Life Sciences, Zhengzhou University, Zhengzhou, Henan 450001, China; ^3^Department of Research and Foreign Affairs, The Affiliated Cancer Hospital of Zhengzhou University, Henan Cancer Hospital, Zhengzhou, Henan 450008, China; ^4^Department of Medicine, Weill Cornell Medical College, New York, NY 10065, USA

## Abstract

**Background:**

This study aims to explore the efficacy of tumor-infiltrating lymphocytes (TIL) along with interferon-*α* (IFN-*α*) to treat stage III malignant melanoma (MM) patients in China.

**Methods:**

Between May 2010 and October 2014, 77 patients of stage III MM who underwent surgery were collected in this study. These patients were divided into two groups: patients who received TIL + IFN-*α* ± RetroNectin-activated cytokine-induced killer cells (R-CIK) in Arm 1 (*n* = 27) and IFN-*α* ± R-CIK in Arm 2 (*n* = 50) as adjuvant therapy. The primary endpoints were disease-free survival (DFS) time and DFS rates measured at time points of 1, 2, and 3 years. The secondary endpoints were overall survival (OS) rates measured at time points of 1, 2, 3, and 5 years as well as OS as evaluated by Kaplan-Meier.

**Results:**

Our results indicated that the median DFS and OS in Arm 1 were significantly better than those in Arm 2. The data also demonstrated that DFS rate and OS rates in Arm 1 were significantly better than those in Arm 2 at all measured time points.

**Conclusion:**

Patients who undergo surgical excision of stage III MM appear to enjoy prolonged DFS and OS when treated with TIL + IFN-*α* compared to IFN-*α* alone.

## 1. Introduction

The epidemiology data of the United States in 2014 indicated that an estimated 76,100 patients were diagnosed with melanoma and 9710 patients died from the disease [1]. Worse yet, incidence of this disease appeared to be rising rapidly. From 2002 to 2006, the incidence of melanoma increased by 33% among men and 23% among women [2]. Currently, definitive surgical excision is still the primary treatment for candidate malignant melanoma patients. However, the rate of relapse for stage III malignant melanoma patients remains very high even with the administration of adjuvant high-dose interferon-*α* (IFN-*α*) [3]. In numerous clinical trials, this IFN-*α* adjuvant therapy has been shown to improve DFS but not OS [4–6]. In cases of metastatic disease, prognosis is exceptionally poor with mOS of 6 to 8 months and a 5-year OS rate of approximately 6% [7, 8]. Recently, numbers of novel immunotherapies such as anticytotoxic T-lymphocyte-associated protein 4 (anti-CTLA-4) and programmed death 1 (anti-PD-1) antibodies have gained FDA approval. While, anti-PD-1 antibody, which has been associated with a 38% objective response rate (ORR), is only approved for advanced malignant melanoma [9]. Therefore, the identification of new postoperative therapies for malignant melanoma patients is of urgent importance.

For a long time, TIL therapy had already shown promise for advanced melanoma patients, with 51% to 72% ORR by Rosenberg et al. [10–14]. Then, more and more clinical trials applied TIL to treat advanced malignant melanoma patients; however, the data of applying TIL to treat postoperative malignant melanoma patients is still few. In 2002, Labarriere et al. reported that TIL treatment combined with interleukin-2 (IL-2) can prolong the DFS of stage III malignant melanoma patients, who emerged only one metastatic lymph node [15]. Unfortunately, in 2007, the 7 years' follow-up data from that same trial failed to show that TIL treatment combined with IL-2 prolonged RFS or OS overall. Intriguingly, however, in patients with only one positive lymph node, the estimated DFS and OS were significantly prolonged from the TIL + IL-2 therapy compared with IL-2 alone therapy [16]. In 2014, this same team of researchers updated their data and reported that TIL therapy can enhance the curative efficacy of patients with low tumor burden [17]. These data suggest that TIL treatment can be effective against malignant melanoma when applied in the right patient population. However, the efficacy of combining TIL therapy with administration of IFN-*α* to treat stage III malignant melanoma is unclear. The aim of our current study is to evaluate the efficacy of adjuvant TIL therapy with IFN-*α* for patients undergoing resection of stage III malignant melanoma.

## 2. Methods

### 2.1. Patients

From May 2010 to October 2014, 77 patients undergoing surgical resection of stage III malignant melanoma were collected in this study. Then, TIL + IFN-*α* ± R-CIK treatment was provided to 27 patients of Arm 1 and IFN-*α* ± R-CIK treatment was provided to 50 patients of Arm 2. This study was approved by the ethics committee at The Affiliated Cancer Hospital of Zhengzhou University, and an approved consent form was signed by all patients. The procedures were in accordance with the Helsinki Declaration of 1975 and Good Clinical Practice guidelines. Although the two groups have different sample sizes, the baselines of the two Arms were relatively well balanced. The detailed baseline of the 77 patients is listed in Table 1.

### 2.2. Retrospective Analysis and Follow-Up

The primary endpoint was DFS, with DFS rates measured at time points of 1, 2, and 3 years. The secondary endpoints were 1-, 2-, 3-, and 5-year OS rates as well as OS as evaluated by Kaplan-Meier analysis, and potential prognostic factors were also analyzed by univariate analysis and multivariate analysis. Following surgery, patients were seen for follow-up every 3 months for a two-year period. During postoperative years 2 to 5, patients were reevaluated every 6 months. Beyond the 5-year mark, follow-up evaluation occurred annually. The follow-up deadline was December 8, 2016. When follow-up evaluation revealed metastatic disease, other therapies were employed, including surgery, immunotherapy, chemotherapy, and radiotherapy (Table 1).

### 2.3. Preparation of TIL

Following surgery, fresh excised tumor tissues were used to culture TILs. Firstly, the excised tumor tissues were sliced into pieces of approximately 2 to 3 mm^3^ in size using a scalpel. Secondly, collagenase, DNase I type IV, and hyaluronidase type V (Sigma-Aldrich, St. Louis, MO, United States) were used to perform enzymatic digestion of these tissues for 2 to 3 hours at room temperature to obtain single-cell suspension. Thirdly, the single-cell suspension was filtered, washed twice with phosphate-buffered saline (PBS), and then incubated in a 12-well plate at a concentration of 1.0 × 10^6^ TIL/ml in X-VIVO medium (Muenchensteinerstrasse 38 CH-4002 Basel, Switzerland) with 7000 IU/ml recombinant human interleukin-2 (rhIL-2). The next day, the cell suspension was removed and further purified via Ficoll gradient. The purified bulk TIL culture was maintained at a concentration of 1-2 × 10^6^ cells/ml in X-VIVO medium with 7000 IU/ml rhIL-2 until all melanoma cells were eliminated and a cell number of at least 5 × 10^7^ TIL cells were achieved. This culture process required approximately 10 to 14 d. Finally, the cultured TIL cells were immediately used with anti-CD3 antibody (GE Healthcare Biosciences, Pittsburgh, PA, USA; 5 *μ*g/ml) and 1000 IU/ml rhIL-2 for large-scale expansion. By this process, cultures were expanded to 5 × 10^9^ TIL cells and were harvested. Finally, these cells were infused back into patients.

### 2.4. Preparation of R-CIK

Peripheral blood mononuclear cells (PBMCs) of the patients were used to culture R-CIK. The detailed process of R-CIK preparation is the same to our published data [18]. Then, at the transfusion day, the dose of R-CIKs is about 5 × 10^9^ cells.

### 2.5. Phenotype Detection

In order to analyze the cell population of TILs before transfusion, they were stained with antibodies against CD3-FITC, CD4-PE-Cy7-A, CD8-APC-Cy7-A, and CD16/CD56-PE (BD Bioscience, San Jose, CA, USA) and flow cytometry was performed using a BD FACSCanto cell sorter (BD Bioscience, San Jose, CA, USA). Finally, the proportion of CD3+CD4+ and CD3+CD8+ cells of TILs was analyzed by gating the CD3+ population, and the percentage of CD3-CD16+ CD56+ cells of TILs was analyzed by CD45+ gating.

### 2.6. Statistical Analysis

Spss17.0 software was used to perform the statistical analysis. The Kaplan-Meier method was used to analyze the DFS and OS. Univariate and multivariable analyses also were used to analyze the prognostic factors. *P* < 0.05 was considered to demonstrate a statistically significant difference.

## 3. Results

### 3.1. Phenotype Analysis

**Figure 1:**
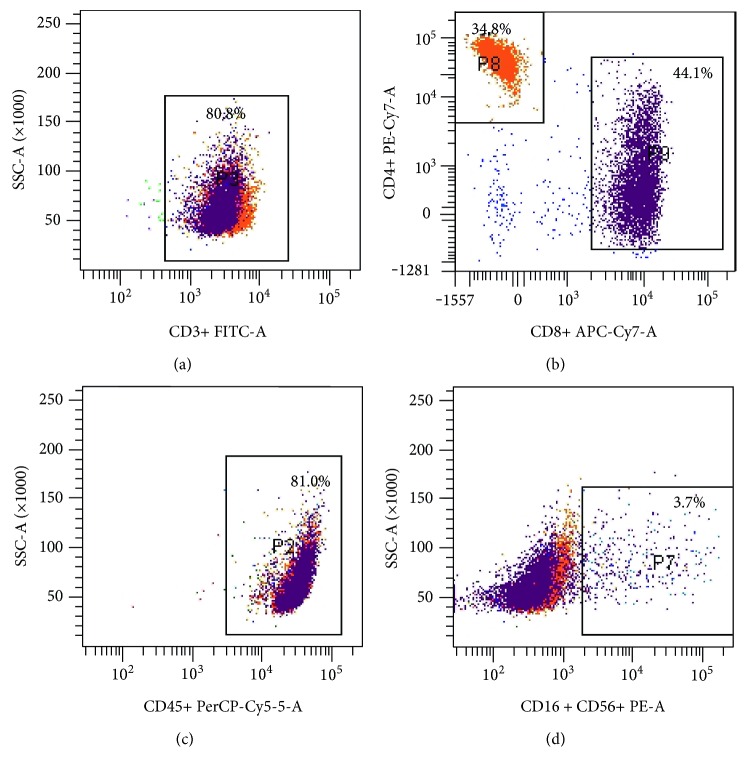
(a) The proportion of CD3+ T cells among TIL cells. (b) The proportion of CD3+CD4+ and CD3+CD8+ T cells among TIL cells. (c) The proportion of CD45+ T cells among TIL cells. (d) The proportion of CD3−CD16+CD56+ T cells among TIL cells.

Before transfusion of TIL cells to patients, we used flow cytometry to detect the proportion of CD3+, CD3+CD4+, CD3+CD8+, and CD3-CD16+CD56+ cells (Figures 1(a), 1(b), 1(c), and 1(d)). When the proportion of CD3+, CD3+CD4+, CD3+CD8+, and CD3-CD16+CD56+ cells reached appropriate levels, then we transfused the TIL back to patients. At the time of delivery of cultured TIL back to patients, the composition of the transfused cells was as follows: CD3+ 80.8% ± 3.23%, CD3+CD4+ 34.8% ± 2.14%, CD3+CD8+ 44.1% ± 2.56%, and CD3-CD16+CD56+ 3.7% ± 0.34%.

### 3.2. Treatment Outcomes

**Figure 2:**
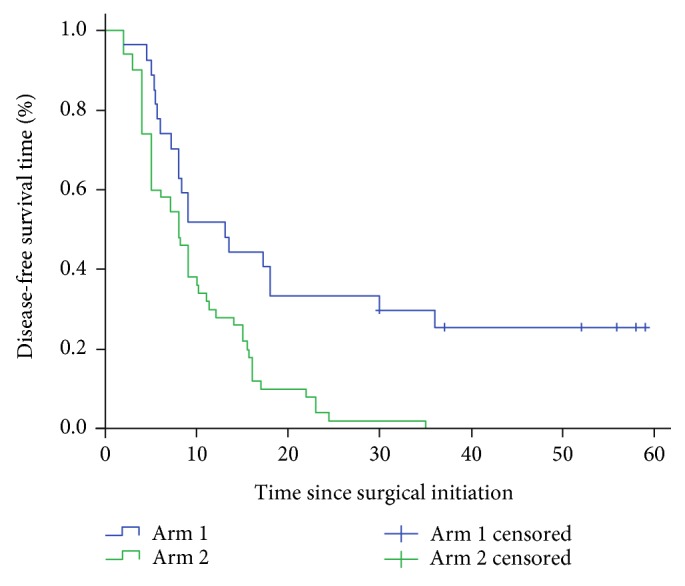
Disease-free survival time of Arm 1 versus Arm 2 was calculated in 27 patients in Arm 1 compared with 50 patients in Arm 2.

**Figure 3:**
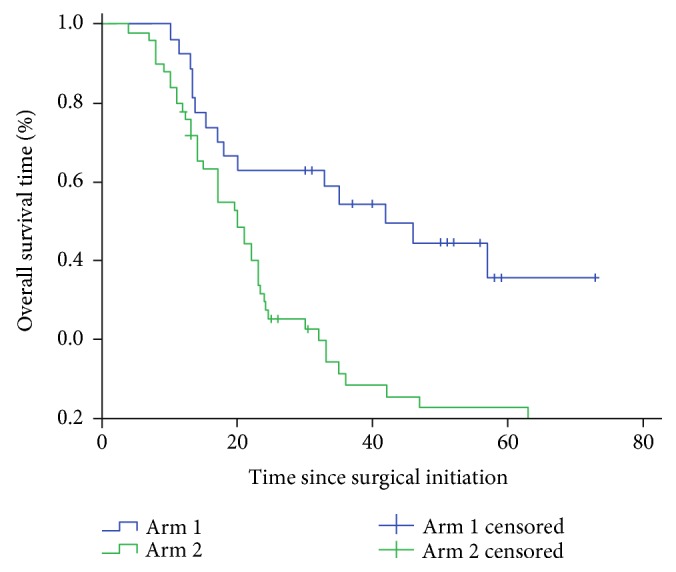
Overall survival time of Arm 1 versus Arm 2 was calculated using 27 patients in Arm 1 compared with 50 patients in Arm 2.

Our data demonstrated that the mDFS and mOS of Arm 1 versus Arm 2 were 23.66 months versus 9.78 months (*χ*^2^ = 11.559, *P* ≤ 0.001, Figure 2) and 43.75 months versus 21.86 months (*χ*^2^ = 15.03, *P* ≤ 0.001, Figure 3), respectively. Then, we also analyzed the 1-year DFS rates and OS rates, 2-year DFS rates and OS rates, 3-year DFS rates and OS rates, and 5-year OS rates. The data indicated that DFS rate and OS rates in Arm 1 were significantly better than those in Arm 2 at all measured time points. The detailed data was listed in Table 2. Thus, it appears that stage III malignant melanoma patients can benefit from TIL + IFN-*α* treatment.

### 3.3. Prognostic Factors of TIL + IFN-*α* ± R-CIK Treatment in Arm 1

The DFS and OS of Arm 1 patients achieved greater improvement compared with those of Arm 2 patients. In order to observe potential prognostic factors in the Arm 1 treatment group, then we analyzed many factors such as sex, age, KPS scores, cell numbers for transfusion, number of culture days, and use of R-CIK therapy. Although univariate analyses indicated that KPS scores, transfused cell numbers, and increased duration of culture were potential predictive factors (Table 3), there were no significant differences by multivariate analysis based on these predictive factors (Tables 4 and 5).

### 3.4. Prognostic Factors of IFN-*α* ± R-CIK Treatment in Arm 2

In Arm 1, our data indicated that adding R-CIK might not improve the DFS and OS of stage III malignant melanoma patients. To investigate whether adding R-CIK can improve the DFS or/and OS of the patients of Arm 2, we also used univariate analysis. Unfortunately, our data demonstrated that there were no significant differences whether with R-CIK therapy or not by univariate analysis (DFS: 9.94 months versus 8.40 months, *P* = 0.707; OS: 21.66 months versus 23.83 months, *P* = 0.770). Thus, it appears that stage III malignant melanoma patients cannot benefit from R-CIK based on IFN-*α* therapy.

### 3.5. Adverse Events

In this retrospective analysis, all patients completed our immunotherapy. There were no severe adverse effects (grade 3 or grade 4) associated with TIL, IFN-*α*, or R-CIK therapy. The primary side effects of immunotherapy (grade 1 or grade 2) were fever, arthralgia, nausea, leucopenia, liver dysfunction, anemia, and vitiligo (Table 6).

## 4. Discussion

Clearly, surgery is still the appropriate primary treatment for candidate malignant melanoma. However, in high-dose IFN-*α* as a current adjuvant therapy, the rate of relapse for stage III malignant melanoma patients remains very high [3]. The treatment of stage III malignant melanoma patients with TIL in combination with IL-2 has previously demonstrated promising results [15–17]. Unfortunately, this combination appears to prolong DFS and OS only among those patients with a single-positive lymph node.

Based upon these encouraging results, efforts to improve the treatment method will be an urgent significance. In our study, administration of cultured autologous TIL combined with IFN-*α* therapy was employed as an adjuvant treatment strategy for stage III malignant melanoma patients. This was compared to the use of IFN-*α* alone. Our data indicates that TIL combined with IFN-*α* therapy can improve the DFS and OS of stage III malignant melanoma patients. From 1991 to present, CIK cell therapy has been applied as an immunotherapy for cancer patients in many clinical trials, including in patients with hepatocellular carcinoma (HCC), non-small-cell lung cancer (NSCLC), and renal cell carcinoma (RCC) [19–26]. And in our Immunotherapy Center of The Affiliated Cancer Hospital of Zhengzhou University, we also did some work to demonstrate that R-CIK (or CIK) combined with chemotherapy or not can prolong the mOS of HCC, RCC, pancreatic cancer, and so on [18, 27–31]. Therefore, R-CIK therapy was often employed in both Arm 1 and Arm 2 patients in order to increase the treatment efficacy. However, in our analysis of Arm 1 and Arm 2 data, there was no difference in DFS and OS with or without R-CIK. Up to now, many experiments indicate that CIK or R-CIK is considered to a nonspecific immunotherapy, which has major histocompatibility- (MHC-) unrestricted cytotoxic effect [26, 32]. Therefore, R-CIK and IL-2 are all nonspecific immunotherapy methods. It appears that combined two nonspecific immunotherapies (R-CIK and IL-2) may not improve the prognosis of stage III malignant melanoma patients.

As we all have known, Rosenberg et al. had done many experiments for metastatic melanoma by applying TIL combined with nonmyeloablative chemotherapy with or without 1200 cGy total body irradiation. The objective response rate can achieve more than 50% [33–35]. In our study, we applied TIL combined with IFN-*α* to treat stage III malignant melanoma as an adjuvant therapy, which indicated that this therapy can prolong the DFS and OS of these patients. Therefore, we conclude that patients diagnosed with stage III malignant melanoma can benefit from TIL + IFN-*α* treatment after surgery.

To explore the prognostic factors governing the efficacy of TIL + IFN-*α* treatment in Arm 1, we analyzed individual results in Arm 1 and correlated them to sex, age, KPS scores, cell numbers at time of transfusion, duration of culture, and presence or absence of R-CIK therapy. Although univariate analyses identified KPS scores, cell numbers for transfusion, and number of culture days as potential predictive factors, there were no significant differences based on these potential predictive factors by multivariate analysis. This leads us to conclude that adjuvant TIL therapy combined with administration of IFN-*α* can prolong DFS and OS in stage III malignant melanoma patients generally. However, adding R-CIK cannot improve the DFS and OS of stage III malignant melanoma patients further. In addition, in our study of all the patients, there were no treatment-related mortalities, and the toxic effects were comparable with previous TIL studies and IFN-*α* studies. While TIL cultures and transfusions require high laboratory expertise, the quality of cultured TIL is the key problem in clinical use. Most of all, our study demonstrated that TIL combined with IFN-*α* might be a good method for stage III malignant melanoma patients. In the future, a multicenter randomized phage study will become a better way to reveal the true clinical contribution of TIL combined with IFN-*α* for the treatment of stage III malignant melanoma.

## 5. Conclusions

In summary, adjuvant adoptive TIL therapy combined with IFN-*α* therapy can prolong the DFS and OS of stage III malignant melanoma patients who undergo surgical excision. Toxicity and side effects were quite manageable. In the future, more studies should be performed to provide additional data regarding the efficacy of adjuvant TIL combined with IFN-*α* therapy in the management of stage III malignant melanoma.

## Figures and Tables

**Table 1:** Clinical characteristics of 77 patients in this study.

	Arm 1 (*n* = 27)	Arm 2 (*n* = 50)	*P* value
Sex			
Male	12	29	0.255
Female	15	21	
Age (year)			
>60	11	21	0.915
≤60	16	29	
KPS			
≥80	22	45	0.289
<80	5	5	
Primary tumor site			
Mucosa type^∗^	4	15	0.140
No-mucosa type^∗^	23	35	
R-CIK			
Yes	22	45	0.289
No	5	5	
Treatment after metastasis			
Surgical	8	10	—
Immunotherapy	27	45	—
Chemotherapy	5	10	—
Radiotherapy	3	6	—
Any 2 or more	16	35	—
Any 3 or more	3	8	0.912

^∗^In Arm 1, mucosa type patients include 3 patients with nasal cavity mucosa melanoma and one patient with mouth cavity melanoma. No-mucosa type patients include 12 patients with acral lentiginous melanoma, 7 patients with nodular melanoma, and 4 patients with superficial spreading melanoma. In Arm 2, mucosa type patients include 5 patients with rectal mucosa melanoma, 6 patients with nasal cavity mucosa melanoma, 2 patients with mouth cavity melanoma, one patient with penis mucosa melanoma, and one patient with vaginal mucosa melanoma. No-mucosa type patients include 14 patients with acral lentiginous melanoma, 11 patients with nodular melanoma, and 10 patients with superficial spreading melanoma.

**Table 2:** Treatment group outcomes.

Treatment group	DFS			OS		
	Arm 1	Arm 2	*P*	Arm 1	Arm 2	*P*
1-year DFS or OS rates	48.21% (95% CI 29.76–64.46%)	28.00% (95% CI 16.45–40.75%)	*0.04*	92.59% (95% CI 73.50–98.09%)	78.00% (95% CI 63.81–87.16%)	*0.03*
2-year DFS or OS rates	33.38% (95% CI 17.80–49.78%)	2.00% (95% CI 0.16–9.23%)	0.00	62.96% (95% CI 42.12–78.07%)	32.00% (95% CI 19.70–44.97%)	0.00
3-year DFS or OS rates	25.96% (95% CI 12.47–41.75%)	0% (95% CI 0-0%)	0.00	55.56% (95% CI 35.22–71.81%)	16.00% (95% CI 7.50–27.37%)	0.00
5-year DFS or OS rates	—	—	—	48.14% (95% CI 28.69–65.19%)	12.00% (95% CI 4.88–22.60%)	0.00

**Table 3:** Univariate analysis.

	DFS (months)	*P*	mOS (months)	*P*
Age (years)				
>60	25.69	0.637	42.79	0.814
≤60	21.27		38.82	
Sex				
Male	20.47	0.560	37.33	0.861
Female	28.17		44.10	
KPS scores				
≥80	26.69	0.032	49.33	0.020
<80	13.27		21.50	
Cell numbers for transfusion				
<8 × 10^9^	6.91	0.000	16.94	0.000
≥8 × 10^9^	30.97		53.46	
Culture days				
<30	30.77	0.014	55.78	0.001
≥30	13.79		24.43	
R-CIK				
Yes	29.22	0.578	44.29	0.791
No	22.35		35.28	

**Table 4:** Multivariate analysis (DFS).

Parameters	Hazard ratio	95% confidence interval	*P* value
KPS (≥80 scores versus <80 scores)	0.948	(0.889–1.011)	0.104
Cell numbers for transfusion (≥8 × 10^9^ versus <8 × 10^9^)	0.912	(0.782–1.064)	0.276
Culture days (<30 days versus ≥30 days)	1.038	(0.976–1.105)	0.268

**Table 5:** Multivariate analysis (OS).

Parameters	Hazard ratio	95% confidence interval	*P* value
KPS (≥80 scores versus <80 scores)	0.944	(0.878–1.015)	0.119
Cell numbers of transfusion (≥8 × 10^9^ versus <8 × 10^9^)	0.794	(0.586–1.075)	0.136
Culture days (<30 days versus ≥30 days)	1.064	(0.981–1.153)	0.134

**Table 6:** Distribution of adverse events.

Side effects	Arm 1		Arm 2	
	Grade 1/2	Grade 3/4	Grade 1/2	Grade 3/4
Fever	13	0	20	0
Arthralgia	5	0	13	0
Nausea	3	0	12	0
Leukopenia	4	0	8	0
Liver dysfunction	2	0	9	0
Anemia	3	0	10	0
Vitiligo	2	0	1	0

## Abbreviations

IFN-*α*: Interferon-*α*
DFS: Disease-free survival time
OS: Overall survival time
TIL: Tumor-infiltrating lymphocytes
R-CIK: RetroNectin-activated cytokine-induced killer cells
ORR: Overall response rate
ACT: Adoptive cell transfer
PBMC: Peripheral blood mononuclear cells.
